# Ischemia-Modified Albumin as a Marker of Acute Coronary Syndrome: The Case for Revising the Concept of “N-Terminal Modification” to “Fatty Acid Occupation” of Albumin

**DOI:** 10.1155/2017/5692583

**Published:** 2017-03-05

**Authors:** Ismail Oran, Bulent Oran

**Affiliations:** ^1^Department of Radiology, Division of Interventional Radiology, Ege University Medical School, 35100 Izmir, Turkey; ^2^Department of Pediatrics, Division of Paediatric Cardiology, Selcuk University Medical School, 42075 Konya, Turkey

## Abstract

Ischemia-modified albumin (IMA) is assumed “N-terminal modified” albumin which is generated immediately following myocardial ischemia. The diagnosis of IMA is based on reduced cobalt binding affinity to albumin which is attributed mainly to incapability of cobalt to bind at albumin's modified N-terminus. Although the albumin cobalt binding test was accepted as a potentially powerful marker for discriminating acute coronary syndrome from nonischemic chest pain, its usefulness has been brought into question in recent years. Patients with acutely ischemic myocardium exhibit a rapid increase in serum levels of fatty acids (FAs). Almost all released FAs are strongly bound to albumin which create conformational changes in the protein with resultant reduced cobalt binding affinity. There is a clear metabolic and temporal relationship between IMA measured via albumin cobalt binding testing and serum levels of FAs. In line with what has been suggested recently in the literature, we conclude that a shift from the concept of “N-terminal modified” to “FA-occupied” albumin is required, as this better describes IMA in patients with acute coronary syndrome. We also offer “oxidation modified albumin, OMA,” which is conceptually different from the “FA-occupied” IMA, to describe modification of albumin in chronic disease associated with increased oxidative stress.

## 1. Introduction

Acute coronary syndrome is diagnosed biochemically by measuring myocardial proteins in serum originally found in cytoplasm, which appear in the blood not earlier than 4–6 hours after disruption of the myocardial cell membrane. These proteins include creatine kinase MB (CK-MB) and troponin. Biochemical markers that are sensitive and/or specific to ischemia prior to cell damage are therefore of great clinical importance. Such serum-based biochemical test was proposed by Bar-Or [1, 2]. The basic principle of this test involves the N-terminal region of human albumin and its inherent affinity for the cobalt metal ion (the so-called albumin cobalt binding, or ACB assay), the premise being that during myocardial ischemia, the albumin cobalt binding affinity is reduced due to an N-terminal modification of albumin [1, 2]. Note that N-terminal modified albumin has also been termed ischemia-modified albumin (IMA) since Bar-Or's first description.

## 2. Albumin Cobalt Binding (ACB) Assay

The ACB assay was approved by the FDA in 2003 as a method for identifying myocardial ischemia in patients admitted to the emergency department. In essence, the test involves adding cobalt chloride (approximately 1.5 equivalents per albumin molecule) to a serum sample, gently mixing, and then incubating to allow albumin cobalt binding. Dithiothreitol (DTT: a cobalt chelator) is added as a colorizing agent, and the brown color produced by the DTT-cobalt chelation (either free or unbound) is measured at 470 nm using a spectrophotometer. A serum-cobalt blank with no DTT is used for comparison, and the results are presented in absorbance units (ABSU). The ABSU data provide a measure of the concentration of (free or unbound) chelated cobalt in the sample and reflect indirectly the level of IMA; that is, albumin that is incapable of binding cobalt due to what is referred to as “N-terminal modification.”

Although the ACB assay has had FDA approval for more than a decade, the test has not achieved the expected clinical success. We have carried out a literature survey of reports accumulated over the 15 years following the first description of the concept of “N-terminal modification” of albumin to investigate the reasons for the limited reproducibility and accuracy of the ACB assay. We then go on to describe a new concept in the light of this knowledge.

## 3. Deficiencies of ACB Assay

The following list is based on literature reports to date and summarizes the main limitations of the ACB assay.

1. Bar-Or's ACB assay was based on reduced cobalt binding affinity for albumin due to modification of the specific cobalt binding site (i.e., the N-terminal site). However, we now know that albumin has two additional cobalt binding sites, which have a higher affinity than the N-terminus (i.e., site A and site B), as well as an additional fourth binding site (i.e., Cyt-34) [3–6]. In the ACB assay, cobalt is exogenously added at a ratio of 1.5 equivalents per albumin molecule. If this cobalt binds to albumin, it is likely to bind predominantly at site A and/or site B. In other words, the ACB assay measures largely the level of binding of cobalt at site A and/or site B, rather than at the N-terminus; therefore, ischemia-induced N-terminal modification (if present) would lead to only an insignificant effect (or possibly no effect at all) on the total albumin cobalt binding capacity.
2. Bar-Or's ACB assay was based on a protein structural modification (e.g., deletion of the amino acid sequence NH_2_-Asp-Ala-His-Lys) on the albumin N-terminal; that is, so-called ischemia-modified albumin (IMA). However, structural modifications to the N-terminal have not been demonstrated in samples of patients following myocardial ischemia. For example, an analysis of the N-terminal amino acid sequence found a wild-type N-terminal sequence in six of seven patients with a positive ACB assay [7]. Bar-Or himself, the originator of the concept of “N-terminal modification” of albumin, was also unable to demonstrate truncated N-terminal IMA in patients with acute myocardial ischemia [8]. Therefore, and in contrast to Bar-Or's original assumption, IMA may not necessarily represent N-terminal modified albumin.
3. Human albumin has a serum half-life of approximately 20 days, and so N-terminal modified IMA diagnosed via ACB assay should be detectable for several days following myocardial ischemia. However, studies have shown that the level of IMA increases within minutes of the onset of ischemia, remains elevated for 6–12 hours, and then returns to a normal level within 24 hours [9, 10]. The long half-life of albumin, together with the rapid normalization of modified albumin levels following ischemia, does not match the description of N-terminal modified IMA. Rapid clearance of N-terminal modified albumin from the circulation is a possible explanation for this [8]; however, there exists no credible evidence to support this hypothesis. Therefore, it appears that IMA may not represent N-terminal modified albumin.
4. If the N-terminal site was responsible for the cobalt binding as measured by the ACB assay, we would expect a strong relationship between the enzyme-linked immunosorbent assay (ELISA) developed specifically to detect N-terminal modification of albumin and the classical ACB assay in clinical practice. However, Oh et al. [11] found no positive correlation between the ACB assay and the ELISA test for patients with either acute coronary syndrome or nonischemic chest pain. It therefore appears that the N-terminal site of albumin has only a limited (or even insignificant) role in the ACB assay.
5. Domenicali et al. [12] reported posttranscriptional changes of serum albumin in healthy donors as well as in patients with stable cirrhosis and cirrhosis with acute worsening symptoms. They found that all three groups had the same fraction of N-terminal truncated albumin in their serum (approximately 2.5%). However, IMA measured by ACB assay was significantly higher in patients with acute-on-chronic liver failure [13]. This study does not support Domenicali's findings and therefore IMA detected by ACB assay might not be associated with an N-terminal modification only.
6. In Bar-Or's ACB assay, the cobalt chelator DTT was used as a colorizing agent. DTT is also a known thiol-based protein disulfide (S-S) reducing agent, leading to disulfide-bond cleavage and subsequent unfolding of the molecule (i.e., denaturation), when used at concentrations in the range 1–10 mM [14]. In a typical ACB assay, the concentration of DTT is 1.6 mM. Since DTT incubation with subsequent spectroscopic reading ends up within 2 minutes during ACB assay, the denaturation, if present, might be limited in magnitude. It is worth noting, however, that protein denaturation may result in displacement of cobalt from the albumin, leading to reduced cobalt binding capacity, thus impairing the results of the ACB assay.
7. Bar-Or [8] himself questioned the original concept of the ACB assay, based on observations (some of which are listed above) that were not easily explained by the concept of N-terminal modification of albumin. The authors preincubated serum samples with an excess of cobalt, allowing for full saturation of the albumin, and then filtered the albumin to exclude other serum-born proteins capable of binding to cobalt. They washed the remaining albumin solution several times to exclude unbound cobalt and added DTT to the sample. Interestingly, the brown coloration was still present, leading to speculation that DTT-albumin complexes were associated with displacement of cobalt from albumin during the ACB assay [8]. This observation further supports the idea that, as discussed above, the assay DTT itself may denature the albumin in the sample, resulting in displacement of cobalt from the albumin, reducing the cobalt binding capacity of the albumin, and hence impairing the results of the ACB assay.

## 4. Interaction between Fatty Acids and Albumin

Human serum contains a mixture of at least six FAs, as follows (with approximate percentages): oleic acid (38%), palmitic acid (25%), linoleic acid (22%), stearic acid (10%), arachidonic acid (3%), and linolenic acid (2%). Almost all blood FAs are strongly bound to albumin and are transported throughout the body in this form, whereas only a very small percentage (less than 1/100,000) is present in the unbound form, so-called unbound or free FAs [15]. Human serum albumin has at least seven binding sites, with varying affinities for medium- and long-chain FAs [16]. Under normal physiological conditions, on average 0.1–2 molecules of FA are bound to each albumin molecule; however, the molar ratio of FAs to albumin can reach up to 6-7 during fasting or extreme exercise or in patients with liver and cardiovascular diseases (e.g., acute myocardial ischemia) [17, 18]. The numbering of these seven sites (FA sites 1–7) in the crystal structure of albumin is arbitrary and not based on the affinity for FA molecules. Among the seven binding sites on albumin, sites FA2, FA4, and FA5 have been identified as having the highest affinity for FAs [19]. Although the binding pockets appear well adapted to accommodate FA molecules, they are not specific to any particular FAs and thus are capable of binding to other ligand molecules. Fujiwara and Amisaki [20] have recently summarized the list of competing ligands that share common binding site with FAs.

## 5. Fatty Acid as a Marker of Myocardial Ischemia

Myocardial ischemia leads to a hyperadrenergic state within minutes of the onset of chest pain, which results in the breakdown of tissue and plasma phospholipids, as well as triglycerides, resulting in increased plasma concentration of free FAs. Patients with acutely ischemic myocardium exhibit a rapid increase in serum levels of free FAs, which can exceed normal average values at the time of admission by a factor of 3–10 [22–27]. Measurement of serum levels of free FAs in the diagnosis of acute myocardial ischemia has been the subject of a number of patent applications [28–30]. Serum levels of free FAs have also been reported to increase within 30 minutes of coronary balloon angioplasty (a well-known in vivo model for transient myocardial ischemia caused by balloon inflation) and a mean 5-fold increase in FA levels has been reported [31]. A recent multicenter study investigated the utility of measuring levels of free FAs compared with other available clinical tests (i.e., amino terminal pro-B-type natriuretic peptide, IMA, heart fatty acid binding protein, classical troponin T, and high-sensitive troponin I) [32] and found that free FA had the highest overall sensitivity (75%), specificity (72%), and negative predictive values (92%) for discriminating acute coronary syndrome from nonischemic chest pain in patients admitted to the emergency department. Thus, current data, although limited, suggest that monitoring of levels of free FAs in patients presenting with chest pain may provide an early indication of myocardial ischemia that is able to discriminate between acute coronary syndrome and nonischemic chest pain.

## 6. Link between the ACB Assay and Free FA

The first study into the effects of FAs on metal binding to albumin appeared in 2003. A British team found that cadmium binding was dramatically affected by high FA loading of albumin [33]. Mothes and Faller [3] speculated a putative relationship between FA binding to albumin site A and increased levels of free cobalt (i.e., increased IMA) in the ACB assay for patients with myocardial ischemia. Bhagavan et al. [34] reported an in vitro study, using pooled sera, and found a significant positive correlation between free FA and IMA measured by ACB assay; that is, the addition of FAs to the sample resulted in increased IMA levels (i.e., decreased albumin cobalt binding) with the same ratio. Lately, the same team from UK has made substantial progress in understanding the impact of FAs on the binding of albumin to metal ions (i.e., cobalt) and demonstrated allosteric inhibition of cobalt binding with albumin due to FAs. They concluded that IMA may correspond to albumin with increased levels of bound FAs [5, 35, 36].

A number of clinical studies have also discussed the possible links between serum levels of free FAs and the IMA results. Amirtharaj et al. [37] reported an inversely proportional relationship between the albumin cobalt binding capacity and FA level in patients with increased free-FA levels due to fatty liver disease. Bhagavan et al. [34] found an increase in IMA (lower cobalt binding capacity) in the sera of patients with acute myocardial infarction and discussed a plausible relationship between increased levels of free FA and increased IMA as measured by ACB assay. Jalan et al. [13] demonstrated a strong correlation between FAs bound to the FA1 and/or FA2 sites of albumin and reduced cobalt binding capacity and speculated overlap between cobalt binding sites and the FA1 and/or FA2 sites of albumin.

**Figure 1:**
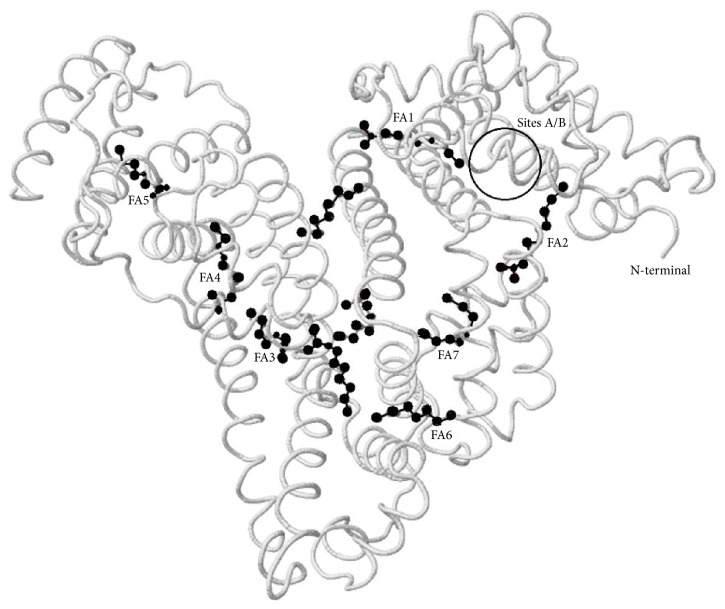
Location of the seven major FA-binding sites of human serum albumin, in relation to the main cobalt binding sites (i.e., N-terminal, site A, and site B). FA2 is one of the high affinity sites for FAs and communicates allosterically with cobalt binding to sites A/B. N-terminus never involves FA binding, and thus continues cobalt binding in the presence of “FA-occupied albumin.” Image was generated using Protein Data Bank (PDB) ID: 1E7E [21] and modified according to Lu et al. [5].

It is unsurprising to see such parallelism between IMA and albumin sites occupied by (bound) FAs, since all cobalt binding sites, except the N-terminal (including site A and site B), share common/neighbour albumin binding sites with FAs. It therefore appears reasonable to suppose that reduced cobalt binding to albumin, which is termed IMA, is actually due to occupation of the metal binding regions at site A and/or site B by bound FAs. The N-terminal site of albumin does not bind to FAs [16, 19, 20, 36] and so “FA occupation” does not nullify completely the total cobalt binding capacity of albumin due to the (unoccupied) N-terminal site, further supporting the general theory of “albumin occupied by FAs” (Figure 1).

## 7. Evidence in Support of “FA Occupation of Albumin”

Elevated levels of free and bound FAs are known to be associated with a number of clinical conditions in addition to myocardial ischemia, including metabolic syndrome, nonalcoholic fatty liver, obesity, cancer, diseases with chronic inflammation, diabetes mellitus, hypertension, cardiovascular disease, stroke, and Alzheimer's disease [38, 39]. Noncardiac sources of increased IMA are associated with ischemic stroke, intracranial haemorrhage, mesenteric ischemia, skeletal muscle ischemia, peripheral atherosclerosis, hyperlipidemia, obesity, metabolic syndrome, hepatosteatosis, preeclampsia, foetal distress, diabetes mellitus, advanced renal disease, and liver cirrhosis [40, 41].

Here we assume, in line with the recent literature, that IMA is in fact “FA-occupied albumin” in patients with acute myocardial ischemia. The preceding section discusses substantial direct and indirect evidence from in vitro and clinical studies, which support the “occupation” concept; however, some additional data are required to support this theory. Some such evidence is discussed below.

1. Other diseases, or clinical or laboratory conditions whereby albumin is “enveloped” by ligands or adducts, further support the “FA-occupied albumin” concept. One such example is poorly controlled diabetes, whereby the nonenzymatic covalent attachment of glucose molecules to albumin, as well as the subsequent oxidation, gives rise to advanced glycation end-products that bind to the albumin surface (also termed “glycated albumin”). The N-terminus itself, 59 lysine and 24 arginine residues on the albumin molecule, acts as potential sites for the formation of glycation products, which impair the ligand binding functions of the protein [42]. If the glycated albumin was occupied by irreversibly bound glycation end-products, we would expect higher levels of IMA due to the inability of cobalt to bind to these (blocked) sites. Indeed, diabetics exhibit higher levels of IMA than control subjects; there is a significant correlation between IMA and HbA_1c_, and patients with poor glycaemia control have higher IMA levels in comparison with those with good glycaemia control [43, 44]. Baraka-Vidot et al. [45] recently reported an in vitro model of glycated albumin, showing a positive correlation between the extent of glycation of albumin and the IMA levels measured by ACB assay (note that there were no other effects that could have potentially led to modifications of albumin other than glycation). Additionally, if the glycated albumins were fully occupied by irreversibly bound glycation end-products, then we would expect higher levels of free FAs, together with lower albumin FA-binding capacity because of the inability of the FAs to bind to the (irreversibly blocked) sites on the albumin. Unsurprisingly, glycated albumin has been shown to exhibit a diminished albumin FA-binding capacity [46, 47]. Thus, the impaired function of glycated albumin further supports the “FA occupation” concept.
2. Human serum albumin is the most abundant circulating protein in the plasma and exhibits important antioxidant activities. Similar to the mechanism for generating “glycated albumin,” the presence of long-standing oxidative stress may result in irreversible adducts on the protein, generating “oxidized albumin,” in particular, thiol oxidation at Cys34 and carbonylation of several amino acids including proline, arginine, lysine, threonine, tyrosine, and methionine [48]. Oxidative damage to albumin impairs the functioning of ligand binding sites [49] and is associated with chronic diseases including diabetes mellitus, nephrotic syndrome, advanced liver diseases, chronic kidney disease, cardiovascular diseases and ageing, Alzheimer's disease, rheumatologic diseases, and cancers associated with increased oxidative stress [48]. If the oxidized albumins were occupied by irreversibly attached oxidation end-products that impair the ligand binding capacity, we would expect to find elevated levels of IMA in such chronic diseases. Anyway, as mentioned above, chronic diseases are associated with increased IMA levels [40, 41]. Thus, the impaired functioning of oxidized albumin also supports the “occupation” concept.
3. If we assume that IMA is actually “FA-occupied” rather than “N-terminal modified” albumin in patients with acute myocardial ischemia, then we would expect the diagnostic accuracy of tests that measure serum levels of FAs to be higher than that of IMA measured by ACB assay. Furthermore, we would also expect tests that measure serum levels of FAs to be useful for discriminating acute coronary syndrome from nonischemic chest pain. Some recent studies with relatively large (>300 patients) sample sizes have reported negative results, calling into question the use of IMA measured by ACB assay as a marker for acute coronary syndrome [32, 50, 51]. Overall, the sensitivity of IMA measured by ACB assay was in the range 60–98%, with specificity in the range 35–94%, and with a negative predictive value of 60–90%. Today, it is generally accepted that the test is useful for ruling out acute coronary syndrome in clinical conditions, with negative troponin and negative ECG [52]. Studies on the use of serum levels of free FAs as a marker for myocardial ischemia are scarce and were discussed in the previous two sections. Although the level of free FAs appears to be more valuable than IMA for identifying acute myocardial ischemia (there are, at least, no studies reporting even insignificant increases in levels of free FAs, in contrast to the reports on IMA), there has been only one study comparing its merit with that of other markers of ischemia [32]. This was a multicenter study, with a total of 318 patients, which evaluated the power of free-FA levels, along with other available clinical tests (i.e., amino terminal pro-B-type natriuretic peptide, IMA, heart fatty acid binding protein, classical troponin T, and high-sensitive troponin I). They found that the free-FA level had the highest overall sensitivity (75%), specificity (72%), and negative predictive value (92%) for discriminating acute coronary syndrome from nonischemic chest pain in patients admitted to the emergency department. In summary, although further study is required, it appears that levels of free FAs have better discriminating power than IMA measured by ACB assay in patients presenting at the emergency department with acute chest pain and suspected acute coronary syndrome. This further supports the concept of “FA occupation.”
4. If IMA is in fact “FA-occupied” rather than “N-terminal modified” albumin and if the ACB assay actually measures the cobalt binding capacity of albumin (which is occupied by FAs in patients with acute myocardial ischemia), then we would expect a strong temporal relationship between IMA and levels of free FAs. Indeed, both IMA and level of free FAs increase within minutes from the onset of ischemia (including balloon-induced myocardial ischemia during percutaneous coronary intervention) [31, 53], remain elevated for 6–12 hours, and then return to normal within 24 hours [27, 40]. This relationship also supports the concept of “FA occupation.”

## 8. Why Should We Make Changes to the Concept of IMA?

As discussed above, there is no positive correlation between the ELISA test developed specifically to detect N-terminal modification of albumin and the classical ACB assay [11]; therefore, the N-terminal site of albumin has no significance with respect to the results of the ACB assay for patients with suspected acute coronary syndrome. Furthermore, if we assume that IMA is “FA-occupied” albumin rather than “N-terminal modified” albumin in patients with acute myocardial ischemia, then we conclude that IMA measured via an ELISA assay is useless for discriminating acute coronary syndrome from nonischemic chest pain. Researchers dealing with IMA-guided diagnosis of acute myocardial ischemia must take this into consideration and should continue to use the classical ACB assay in preference to an ELISA assay to detect IMA which is actually not “N-terminal modified” but “FA-occupied” albumin.

Researchers dealing with chronic diseases associated with “glycated/oxidized” albumin may continue to use both the classical ACB assay and ELISA tests for determining IMA levels, since irreversible modification of the N-terminus (as well as many other moieties of albumin, including cobalt binding sites) is common in such circumstances, making both ACB and ELISA tests useful. Alzheimer's disease is one such well-defined example of chronic disease for which long-standing oxidative stress is significant in the pathogenesis, leading to enhanced protein oxidation, DNA oxidation, lipid peroxidation, advanced glycation end-products, and carbonylated proteins. As expected, IMA levels measured by both classical ACB assay [54] and ELISA [55] were significantly higher in Alzheimer's patients and correlate well with other oxidative stress markers.

A marker like IMA that is sensitive and/or specific to myocardial ischemia prior to cell damage may be enormously valuable to the emergency physician assessing chest pain patients. We still require, however, a better understanding of this marker before it is ready for prime time use. All researchers interested in IMA should be aware of what exactly the IMA is, as well as the difference between the results of the ACB assay and ELISA testing, and what the results of the tests actually mean. We must therefore revise the concept of IMA.

## 9. Conclusion

Despite its deficiencies, the ACB assay for IMA levels may still be useful in emergency settings to discriminate acute coronary syndrome; however, the ELISA test should not be used for this purpose. We conclude that a conceptual change from “N-terminal modified” to “FA-occupied” albumin is required to better delineate IMA in patients with acute coronary syndrome. In that respect, we would like to offer using the new term “oxidation modified albumin” (OMA) instead of well-known “ischemia-modified albumin” to better delineate modified albumin in patients with chronic disease associated with increased oxidative stress. Thus, the new nomenclature may be useful in differentiating albumin modification occurring in acute coronary syndrome with myocardial ischemia from chronic disease with increased oxidative stress. The myocardial ischemia generates “reversible” “ischemia-modified albumin, IMA” secondary to FA occupation of albumin, while the oxidative stress generates “irreversible” “oxidation modified albumin, OMA” secondary to oxidation adducts on albumin.
